# *Ginger *DNA transposons in eukaryotes and their evolutionary relationships with long terminal repeat retrotransposons

**DOI:** 10.1186/1759-8753-1-3

**Published:** 2010-01-25

**Authors:** Weidong Bao, Vladimir V Kapitonov, Jerzy Jurka

**Affiliations:** 1 Genetic Information Research Institute, Mountain View, CA, USA

## Abstract

**Background:**

In eukaryotes, long terminal repeat (LTR) retrotransposons such as *Copia, BEL *and *Gypsy *integrate their DNA copies into the host genome using a particular type of DDE transposase called integrase (INT). The *Gypsy *INT-like transposase is also conserved in the *Polinton/Maverick *self-synthesizing DNA transposons and in the 'cut and paste' DNA transposons known as *TDD-4 *and *TDD-5*. Moreover, it is known that INT is similar to bacterial transposases that belong to the IS*3*, IS*481*, IS*30 *and IS*630 *families. It has been suggested that LTR retrotransposons evolved from a non-LTR retrotransposon fused with a DNA transposon in early eukaryotes. In this paper we analyze a diverse superfamily of eukaryotic cut and paste DNA transposons coding for INT-like transposase and discuss their evolutionary relationship to LTR retrotransposons.

**Results:**

A new diverse eukaryotic superfamily of DNA transposons, named *Ginger *(for '*Gypsy *INteGrasE Related') DNA transposons is defined and analyzed. Analogously to the IS*3 *and IS*481 *bacterial transposons, the *Ginger *termini resemble those of the *Gypsy *LTR retrotransposons. Currently, *Ginger *transposons can be divided into two distinct groups named *Ginger1 *and *Ginger2/Tdd*. Elements from the *Ginger1 *group are characterized by approximately 40 to 270 base pair (bp) terminal inverted repeats (TIRs), and are flanked by CCGG-specific or CCGT-specific target site duplication (TSD) sequences. The *Ginger1*-encoded transposases contain an approximate 400 amino acid N-terminal portion sharing high amino acid identity to the entire *Gypsy*-encoded integrases, including the YPYY motif, zinc finger, DDE domain, and, importantly, the GPY/F motif, a hallmark of *Gypsy *and endogenous retrovirus (ERV) integrases. *Ginger1 *transposases also contain additional C-terminal domains: ovarian tumor (OTU)-like protease domain or Ulp1 protease domain. In vertebrate genomes, at least two host genes, which were previously thought to be derived from the *Gypsy *integrases, apparently have evolved from the *Ginger1 *transposase genes. We also introduce a second *Ginger *group, designated *Ginger2/Tdd*, which includes the previously reported DNA transposon *TDD-4*.

**Conclusions:**

The *Ginger *superfamily represents eukaryotic DNA transposons closely related to LTR retrotransposons. *Ginger *elements provide new insights into the evolution of transposable elements and certain transposable element (TE)-derived genes.

## Background

All transposable elements (TEs) can be divided into two major classes: retrotransposons and DNA transposons. Based on their transposition mechanisms, eukaryotic retrotransposons can be further divided into non-long terminal repeat (LTR) retrotransposons and LTR retrotransposons [1]. The latter include five clades: *Copia*, *BEL*, *Gypsy*, endogenous retroviruses (*ERV*) and *DIRS*. DNA transposons in eukaryotes can be divided into 'cut and paste' transposons, self-replicating transposons (*Polinton/Maverick*), rolling circle transposons (*Helitron*), and tyrosine recombinase transposons (*Crypton*) [2,3]. *Cryptons *were originally identified in fungi [4], and recently they were found in sea anemone (*Nematostella vectensis*), sea urchin (*Strongylocentrotus purpuratus*) [5] and insects [6,7].

All known 'cut and paste' DNA transposons consist of 17 superfamilies [2,3,8]. Each superfamily encodes a superfamily-specific transposase (TPase), which is generally referred to as DDE transposase for the universally conserved catalytic amino acids it contains: two aspartic acids (D) and one glutamic amino acid (E). The *Copia*, *BEL*, *Gypsy *and *ERV *LTR retrotransposons also code for DDE transposases responsible for integration of their cDNA copies into the host genome. The LTR retrotransposon-encoded transposases are similar to each other and are conventionally called integrases. The integrases are significantly related to bacterial transposases of IS*3 *and IS*481 *insertion sequences, but their relationship to bacterial IS*630 *insertion sequences and eukaryotic *Tc1/Mariner *DNA transposons appears to be more distant [9-11]. It has been proposed that integrase-encoding LTR retrotransposons evolved from the combination of a non-LTR retrotransposon and a DNA transposon [1,10,12,13]. Also, *Gypsy *integrase-like TPases have been found in rare eukaryotic DNA transposons, *TDD-4 *and *TDD-5 *[14,15]. However, it is unclear whether these eukaryotic DNA transposons were derived directly from a *Gypsy *LTR retrotransposon or an ancestral DNA transposon. Moreover, *Polinton *DNA transposons also encode a conserved protein similar to the *Gypsy *integrase, and it was suggested that an ancestral *Polinton *DNA transposon arose by recruiting either an LTR retrotransposon integrase or DNA transposase by a virus or linear plasmid [16].

In the present work, we describe a superfamily of cut and paste DNA transposons called *Ginger *(for '*Gypsy *INteGrasE Related'), coding for the transposase similar to *Gypsy *integrases. The *Ginger *superfamily is composed of two distinctive groups, *Ginger1 *and *Ginger2/Tdd*. The *Ginger1 *group is reported in this paper, and *Ginger2/Tdd *represents elements phylogenetically related to the previously reported DNA transposon *TDD-4 *[14].

## Results

### *Ginger1 *DNA transposons

**Figure 1:**
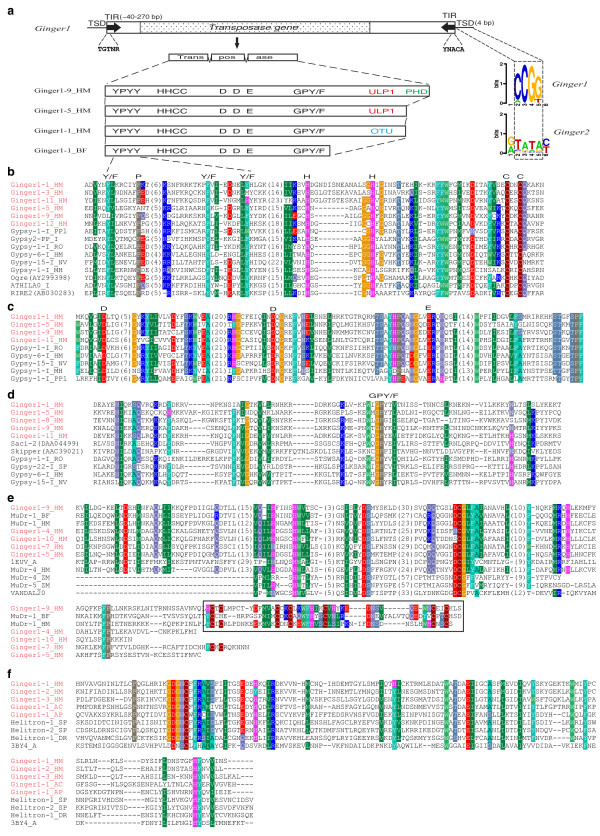
***Ginger1 *(for '*Gypsy *INteGrasE Related 1') elements and transposases**. (a) Schematic features of the *Ginger1 *DNA transposons. Target site duplications (TSDs) and target sequences preferences of the *Ginger1 *and *Ginger2 *groups are compared, which is based on the data of 112 *Ginger1 *elements from *Hydra magnipapillata *and 64 *Ginger2 *elements from *Nematostella vectensis*. (b-d) Alignment of *Ginger1 *and *Gypsy *integrases in the YPYY motif and the H2C2 zinc finger domain (b), the DDE core domain (c), and the GPY/F motif (d). (e) Alignment of the Ulp1 and plant homeodomain (PHD) domains in *Ginger1 *TPases, *Mutator *TPases and yeast protein 1EUV_A; the PHD domain is boxed. (f) Alignment of the ovarian tumor (OTU) domains of *Ginger *TPases, *Helitron *proteins, and yeast protein 3BY4_A.

**Figure 2:**
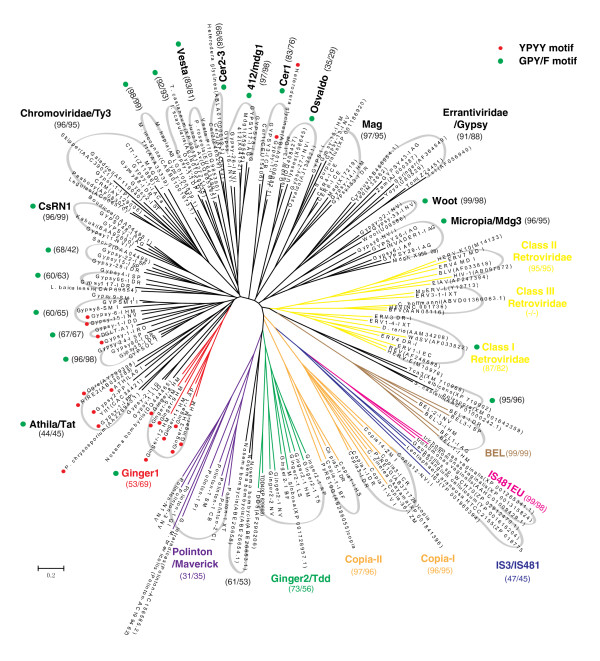
**Phylogenetic relationship between the integrases of *Ginger1 *(for '*Gypsy *INteGrasE Related 1'), *Ginger2 *and *Gypsy *LTR retrotransposons**. The tree, based on the multiple alignments in the zinc finger domain and the DDE domain (see Additional file 1), is constructed by the minimum evolution (ME) method (Poisson correction model, pairwise deletion, gamma parameter = 2, bootstrap replicates = 1,000). The neighbor-joining (NJ) tree is shown in Additional file 2. The ME and NJ bootstrap values of major clades are shown in parenthesis, respectively. Lines in non-black colors differentiate the non-*Gypsy *integrases. The names of known *Gypsy *lineages follow previous literatures [18,20,43,44]. The eukaryotic IS*481*-like integrases in *Trichomonas vaginalis *are designated as IS*418EU*. The two distinct *Copia *clades [45] are named *Copia-I *and *Copia-II*, respectively. Integrases containing the YPYY motif are marked with red dots. The clades containing the GPY/F motif are marked with green dots.

A typical autonomous *Ginger1 *element encodes a single approximately 500 to 800 amino acid long TPase that includes an approximately 400 amino acid N-terminal region highly similar to the integrase (INT) encoded by *Gypsy *LTR retrotransposon (Figure 1). The homologous regions include the H2C2 zinc finger domain, DDE catalytic domain, and the GPY/F motif that mediates multimerization [17]. The latter is a hallmark of *Gypsy *and *ERV *integrases [18]. The amino acid identity between *Ginger1 *TPases and *Gypsy *integrases is up to 40% in the approximately 170 amino acid long DDE catalytic region (Figure 1c). Remarkably, in addition to the domains mentioned above, *Ginger1 *TPases and some *Gypsy *integrases further share an approximate 40 amino acid motif immediately upstream of the zinc finger domain (Figure 1a, b). This motif is not universal in *Gypsy *integrases; it is only present in a limited number of integrases belonging to the *Athila/Tat *group, *Cer1 *group and a few *Athila/Tat *related groups containing *Gypsy-6-I_HM*, *Gypsy-15-I_NV*, *Gypsy-1-I_RO*, *Gypsy-1-I_DD *and *DGLT-A1_I *(Figures 1b and 2). This motif is designated as YPYY for the four conserved amino acids: Y/F-P-Y/F-Y/F (Figure 1b). So far, we have identified dozens of *Ginger1 *families in four animal species, including hydra (*Hydra magnipapillata*), gastropod (*Aplysia californica*), lancelet (*Branchiostoma floridae*) and aphid (*Acyrthosiphon pisum*). The hydra genome harbors the most diverse and abundant *Ginger1 *elements; a total of 12 *Ginger1 *families were identified in this species (Table 1). In the *Ginger1*-*2_HM *family, the divergence of some elements from the consensus is less than 1%, suggesting that *Ginger1*-*2_HM *elements are still active in *H. magnipapillata *genome. In the current release of *B. floridae *genome sequences, *Ginger-1_BF *is found as a single autonomous copy (Table 1), but a few non-autonomous copies carrying the same 5' and 3' end sequences are also found. In the genome of *A. pisum*, only degenerated *Ginger1 *elements are recognizable, but complete elements are identified in the remaining three species. The full length *Ginger1 *elements vary from approximately 2.6 kb to 7 kb, and their terminal inverted repeats (TIRs) are approximately 40 to 270 base pairs (bp) long (Table 1). The target site duplication (TSD) sequences of *Ginger1 *are 4-bp long and the sequences are highly specific: CCGG (75%) or CCGT/ACGG (24%) (Figure 1a). The 5' ends of *Ginger1 *elements show the same conserved TGTNR pattern as those of *Gypsy *LTR retrotransposons.

**Table 1:** *Ginger1 *DNA transposons.

Family	Accession no. and coordinates	Approximate copy number	Length (bp)	TIR length (bp)	Intron number	TPase C-terminal domain
*Ginger1-1_HM*	ABRM01007893.1(3098-6531)	>70	3,425	270	1	OTU
*Ginger1-2_HM*	ABRM01012112.1(11743-14806)	>100	3,064	126	1	OTU
*Ginger1-3_HM*	ABRM01024346.1(3521-874)	>74	2,649	142	1	OTU
*Ginger1-4_HM*	ABRM01000174.1(14295-8442)	>69	5,882	70	0	Ulp1
*Ginger1-5_HM*	ABRM01005903.1(8144-1149)	>40	7,091	45	4	Ulp1
*Ginger1-6_HM*	ABRM01005903.1(15631-10432)	>18	5,214	44	2	Ulp1
*Ginger1-7_HM*	ABRM01000534.1(9961-3846)	>16	6,088	57	2	Ulp1
*Ginger1-8_HM*	ABRM01022284.1(8912-3479)	>20	5,501	58	2	Ulp1
*Ginger1-9_HM*	ABRM01021331.1(4894-8615)	>22	3,758	120	2	Ulp1, PHD
*Ginger1-10_HM*	Join ABRM01011282.1(16673-21197), ABRM01021532.1(14237-12463)	≥5	6,495	106	0	Ulp1
*Ginger1-11_HM*	ABRM01013794.1(17006-12397)	>23	4,645	109	1	OTU
*Ginger1-12_HM*	ABRM01051013.1(2282-4618)	>5	NA	NA	1	Ulp1
*Giger1-1_AC*	AASC02016817.1(36379-31560)	≥5	4,336	140	2	OTU
*Ginger1-1_BF*	ABEP01037661.1(397-5076)	≥1	4,068	77	0	NA
*Ginger1-1-AP*	Join ABLF01057402.1(1975-5709), ABLF01044749.1(3692-2680)	≥1	NA	NA	4	OTU
*Ginger1-2-AP*	ABLF01023350.1(6513-3321)	≥1	NA	NA	1	OTU

AC = *Aplysia californica*; AP = *Acyrthosiphon pisum*; BF = *Branchiostoma floridae*; bp, base pairs; *Ginger *= '*Gypsy *INteGrasE Related'; HM = *Hydra magnipapillata*; OTU = ovarian tumor (OTU) cysteine protease domain (pfam02338); PHD = plant homeodomain finger motif (smart00249); TIR = terminal inverted repeat; TPase = transposase; Ulp1 = C-terminal catalytic domain of Ulp1 protease (pfam02902).

In addition to the INT domains, extra domains are also found at the C-terminus of all *Ginger1 *TPases with the exception of *Ginger1-1_BF *TPase. These domains include the ovarian tumor (OTU) cysteine protease domain (pfam02338), the C-terminal catalytic domain of Ulp1 protease (pfam02902) and the plant homeodomain (PHD) finger motif (smart00249) from the Conserved Domain Database http://www.ncbi.nlm.nih.gov/sites/entrez?db=cdd (Table 1, Figure 1a, e, f). Either OTU or Ulp1 is present in a particular *Ginger1 *TPase, but not both. *Ginger-1_BF *TPase does not contain these extra C-terminal domains, however, it is not clear whether *Ginger-1_BF *TPase itself lacks these C-terminal domains or this particular single copy element contains an internal deletion. It is also worth noting that some *Ginger1 *TPase encoding sequences are interrupted by 1 to 4 introns (Table 1). Except for the first intron of *Ginger-5_HM *(GC-AG) and the first intron of *Ginger-1_AC *(GT-TG), all these introns conform to the canonical GT-AG intron type [19].

### *Ginger2 *DNA transposons

To our knowledge, only two families of eukaryotic DNA transposons, *TDD-4 *and *TDD-5*, have been reported to code for a *Gypsy*-like integrase. They are present in the protist *Dictyostelium discoideum*, and are characterized by 5-bp TSD [14,15]. Their amino acid identity to *Ginger1 *TPases is only approximately 24% in the DDE catalytic domain. To find out more members of this potentially new group of *Ginger *elements, we used the DDE core domain of the *TDD-4 *TPase as a query in Tblastn or Blastp searches against the available National Center for Biotechnology Information (NCBI) databases http://blast.ncbi.nlm.nih.gov/Blast.cgi. As a result, we identified >6 families of homologous DNA transposons from different metazoan species, including hydra (*H. magnipapillata*), sea anemone (*Nematostella vectensis*), aphid (*A. pisum*), nematode (*Trichinella spiralis*), sea snails (*Littorina saxatilis*) and lancelet (*B. floridae*) (Table 2). All these transposons contain approximately 50 to 180 bp long TIRs and produce 4 bp TSDs, instead of the 5 bp TSDs of *TDD-4 *and *TDD-5*. However, as shown below, all these elements and *TDD-4 *or *TDD-5 *belong to the same group (Figure 2), referred to as *Ginger2*. In the fungus *Malassezia globosa*, we also found a protein named XP_001728957.1 closely related to the *Ginger2 *TPases. Neither of the two 5-kb regions flanking the XP_001728957.1-coding region encodes the ribonuclease H or reverse transcriptase. Therefore, even if the XP_001728957.1-coding region is not flanked by TIRs, we classify it as a *Ginger2-1_MG *DNA transposon vestige (Figure 2) rather than an LTR retrotransposon. Like *Ginger1 *and the vast majority of *Gypsy *LTR retrotransposons, the termini of *Ginger2 *follow the same conserved pattern, TGTNR. However, in contrast with the GC-rich 4-bp *Ginger1 *target sequences, *Ginger2 *elements preferentially target 6-bp AT-rich sequences, RTATAY (Figure 1a). *Ginger2 *TPases contain the H2C2 zinc finger domain and the DDE catalytic domain, but they lack the GPY/F and YPYY motifs present in *Ginger1 *TPases. The lowest pairwise amino acid identity in the DDE catalytic core region of the *Ginger2 *elements is approximately 30%, compared with the 36% identity within the same region of the *Ginger1 *group. This suggests that the *Ginger2 *group is more divergent than the *Ginger1 *group and it is consistent with the observation that *Ginger2 *elements are present in protists, fungi and animals, whereas *Ginger1 *elements were identified only in four animal species.

**Table 2:** *Ginger2 *DNA transposons.

Family	Accession no. and coordinates	Approximate copy number	Length (bp)	TIR length (bp)
*TDD-4*	AAFI02000006.1(356458-352615)	>10	3,839	260
*TDD-5*	AF298206	≥1	3,783	297
*Ginger2-1_AP*	ABLF01002904.1(12456-9572)	>2	2,885	166
*Ginger2-1_TS*	ABIR01000229.1(194562-191746)	>9	2,815	112
*Ginger2-1_HM*	ABRM01019362.1(8091-14753)	>6	6,676	45
*Ginger2-1_BF*	ABEP02002130.1(2458-8567)	≥1	6,110	161
*Ginger2-1_NV*	ABAV01011352.1(106952-104193)	>13	2,751	52
*Ginger2-1N1_NV*	ABAV01001774.1(757-1008)	>50	252	52
*Ginger2-2_NV*	ABAV01024827.1(8200-5455)	>13	2,842	93
*Ginger2-1_LS*	CT027673 (88612-94692)	NA	6,081	180
*Ginger2-1_MG*	AAYY01000016.1(145366-147069)	NA	NA	NA

AC = *Aplysia californica*; AP = *Acyrthosiphon pisum*; BF = *Branchiostoma floridae*; bp, base pairs; *Ginger *= '*Gypsy *INteGrasE Related'; HM = *Hydra magnipapillata*; LS = *Littorina saxatilis*; MG = *Malassezia globosa*; NA = not applicable; NV, *Nematostella vectensis*; TIR = terminal inverted repeat; TS = *Trichinella spiralis*.

### Phylogeny of *Ginger1*, *Ginger2 *and *Gypsy *integrases

To better understand the relationships between *Ginger1*, *Ginger2*, and the *Gypsy *lineages, we performed phylogenetic analyses of a wide collection of integrases from *Gypsy *LTR retrotransposons, exogenous/endogenous retroviruses, and *Polinton/Maverick *DNA transposons. *Copia *and *BEL *integrases and some integrase-like transposases from bacteria and protozoan *Trichomonas vaginalis *were included as outgroups. These bacterial transposases are from the IS*3 *and IS*481 *elements, the protozoan integrase-like TPases are closely related to them. TPase from eukaryotic *Mariner/Tc1 *and *Pogo *elements and bacterial IS*630 *elements are not included for analysis because they are phylogenetically closer to each other [8], and are more distantly related to the integrases encoded by LTR retrotransposons and *Ginger *than those encoded by IS*3 *and IS*481 *elements (data not shown). Although our phylogenetic tree is based on the limited sequence information from the zinc finger domain and the DDE core domain (see Additional file 1), most *Gypsy *integrases are clustered together away from other older groups, such as IS*3*/IS*481*, *Copia*, *BEL *and retrovirus [1], with the exception of two lineages of*Gypsy*-like integrase from fungal species, clustering with *Polinton *group and retrovirus groups, respectively (Figure 2). Except for the *Woot *element being separated from the *Osvaldo *clade, the clades and the polytomy distribution of all known *Gypsy *lineages are consistent with the other studies based on the analysis of multiple domains [20,21]. In addition to the known lineages, some extra *Gypsy *clades also appear in our phylogeny, probably due to a larger data set; some of them might represent new lineages of the *Gypsy *LTR retrotransposons. Remarkably, the *Ginger1 *and *Ginger2 *groups are distinctly separated (Figure 2): *Ginger2 *integrases tend to group with the integrases of *Polinton/Maverick *DNA transposons, while *Ginger1 *are closely grouped with *Athila/Tat *lineage of *Gypsy *LTR retrotransposons. Although the YPYY motif is not included in the sequence information used to build the tree, the majority of YPYY motif-containing *Gypsy *integrases apparently cluster together with the *Ginger1 *TPases that also contain the YPYY motif (Figure 2), indicating the YPYY motif is genetically significant. However, no *Gypsy *lineages are found coclustering with *Ginger1 *or *Ginger2 *TPases with significant bootstrap values. A similar pattern is also observed in the tree constructed using the different neighbor-joining method (see Additional file 2).

### Host genes derived from *Ginger1 *TPases

**Figure 3:**
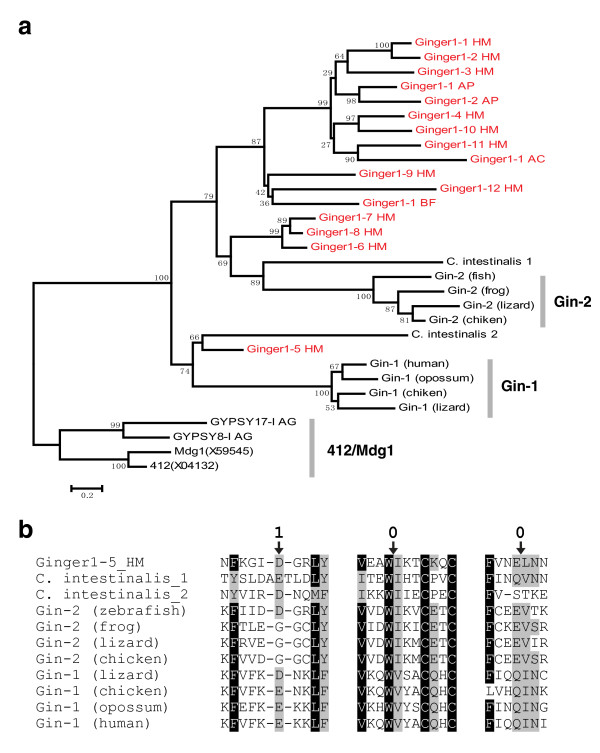
**Host genes evolved from *Ginger1 *(for '*Gypsy *INteGrasE Related 1') DNA transposon**. **(a) **The phylogenetic relationship between *Gin-1*, *Gin-2*, and the *Ginger1 *elements (in red). The tree is constructed by the minimum evolution (ME) method (Poisson correction model, pairwise deletion, gamma parameter = 2, 1,000 bootstrap replicates). The sequences and alignment are shown in Additional file 3. *C. intestinalis_1 *gene refers to XM_002130131.1 gene; *C. intestinalis_2 *gene corresponds to expression sequence tag (EST) named FF869668.1. Integrases from *Gypsy 412/Mdg1 *lineage are included as outgroups. **(b) **The alignment of the local sequences around the three conserved introns in *Ginger1-5_HM *TPase and host proteins. The arrows point to the intron positions, the Arabic numbers (above) indicate the intron phase.

*Gypsy integrase-1 *gene (*Gin-1*), encoding a *Gypsy *integrase-like protein, was thought to have evolved from a *Gypsy *LTR retrotransposon related to the *412/Mdg1 *lineage [22]. However, *Gin-1 *genes and a number of other homologous genes actually evolved from the *Ginger1 *elements. The most parsimonious scenario is that two independent exaptations of *Ginger1 *took place during the evolution of vertebrates, which gave rise to two sets of orthologous genes designated here as *Gin-1 *and *Gin-2 *(Figure 3). The first exaptation event gave rise to *Gin-2 *genes and probably happened in the common ancestor of vertebrates, while the second gave rise to *Gin-1 *genes and happened more recently in the common ancestor of reptiles and mammals. For example, *Gin-1 *genes are present in lizard *Anolis carolinensis *(FG759656.1), chicken *Gallus gallus *(XP_424858.2), opossum *Monodelphis domestica *(XP_001380076.1) and human (NP_060146.2); *Gin-2 *genes were found in fish *Danio rerio *(CAM46974.1), frog *Xenopus tropicalis *(AAI69154.1), lizard *Anolis carolinensis *(FG723791.1) and chicken *Gallus gallus *(XP_415124.1). *Gin-2 *genes are not found in any currently sequenced mammalian genomes and it is likely that *Gin-2 *gene was lost in the early stage of mammal evolution. Consistent with this scenario, *Gin-1 *and *Gin-2 *encoded proteins form two clusters in the phylogenetic tree, and both cocluster within the *Ginger1 *families rather than any other *Gypsy *lineages (Figure 3a, Additional file 3).

Of the six to eight introns in each of the host genes, only three are universally conserved: they are found at the same positions and have the same intron phases (Figure 3b). Strikingly, all the three conserved introns are found in *Ginger1*-*5_HM *TPase gene (Figure 3b), which has four introns in total. In addition, the first conserved intron is also present in the *Ginger1*-6,7,8_*HM *TPase genes (data not shown). The data strongly indicate that *Gin-1 *and *Gin-2 *genes are derived from a *Ginger1*-*5_HM*-like element. In invertebrate tunicate *Ciona intestinalis*, two genes (XM_002130131.1 and FF869668.1) may also be host genes derived from *Ginger1-5_HM*-like elements. They also contain the three conserved introns (Figure 3b), and their upstream and downstream genes are only within the range of approximately 0.5 kb to 2.5 kb, within which no flanking TIRs are found.

## Discussion

In the present work, we report a new eukaryotic superfamily of DNA transposons, named *Ginger*, encoding transposases homologous to the integrases of *Gypsy *LTR retrotransposon. To date, we have identified two distinct *Ginger *groups, *Ginger1 *and *Ginger2/Tdd*. These groups could also be viewed as different superfamilies based on their plausible independent origin. However, further classification is left open here, due to uncertainties inherent in the current phylogenetic data. Previously, *DIRS *and *Ngaro *retrotransposons and *Crypton *DNA transposons were also found to encode the same class of proteins (tyrosine recombinase). However, their evolutionary relationship is not well understood yet due to the scarcity of data [4,23]. Therefore, the relationship between the *Gypsy *LTR retrotransposons and the *Ginger *DNA transposons is of particular interest from the evolutionary perspective.

It is known that transposases from bacterial transposons that belong to the IS*3 *and IS*481 *families are significantly similar to the integrases encoded by eukaryotic LTR retrotransposons [9,11]. Moreover, numerous families of the IS*3 *and IS*481 *transposons are characterized by the 3'-TG and CA-5' termini, and by 3 to 5-bp TSDs, analogous to LTR retrotransposons [9,11]. It has also been proposed that in an early eukaryotic species a first LTR retrotransposon evolved from a non-LTR retrotransposon, which recruited a transposase from a DNA transposon [1,10,12,13]. Given the significant similarity between the integrases encoded by *Ginger1*, *Ginger2 *and various LTR retrotransposons, either the *Ginger1 *or *Ginger2 *groups of transposons, or both, can be viewed as descendants of the ancestral eukaryotic DNA transposons which provided a TPase transformed into the integrase of the first LTR retrotransposon in early eukaryotes. However, as suggested by the modular evolution model of transposable elements [13], the evolutionary scenarios of *Ginger *and LTR integrases may be multifold.

If *Gypsy *was first derived through the fusion of reverse transcriptase with a DNA transposase [1,10,12,13], one possible scenario is a 'reverse evolution' in which *Ginger1 *elements originated from a *Gypsy *LTR retrotransposon. This scenario is based on the remarkable similarity between *Ginger1 *TPase and *Gypsy *INT, plus the fact that *Ginger1 *and *Ginger2/Tdd *are two distinct groups on the phylogenetic tree (Figure 2). *Ginger1 *TPases not only share up to 40% amino acid sequence identity in the DDE region with some *Gypsy *integrases, but also contain a GPY/F domain, which has been found only in *Gypsy *and retroviral integrases so far [18]. Moreover, *Ginger1 *TPases and a subset of *Gypsy *integrases contain the same YPYY motif (Figures 1b and 2). The figure of 40% amino acid sequence identity is comparable to the upper similarity level between integrases from different *Gypsy *lineages (data not shown). In this scenario, the *Ginger2 *elements may resemble the ancestor element that was recruited into the first LTR retrotransposon.

Alternatively, it is still possible that that *Gypsy*, *Copia *and *BEL *LTR retrotransposons arose independently in early eukaryotes by recruiting three different transposases from the same *Ginger*-like superfamily. According to this scenario, *Ginger1 *transposase/transposons may be the 'best preserved' descendants/relatives of the eukaryotic transposon that 'gave birth' to the first *Gypsy*. Another variant of this scenario is that all LTR retrotransposon-encoded integrases arose from the common ancestor of *Ginger1 *and *Ginger2 *TPases. Thus, among numerous hypothetical lineages of this ancestor DNA transposon, *Ginger1 *and *Ginger2 *may be the only lineages that survived from the times of early eukaryotic evolution, which took place over 1.6 billion years ago. According to this scenario, the ancient eukaryotic transposase that transformed into the integrase of the first LTR retrotransposon was likely composed of the H2C2 zinc finger, GPY/F motifs and YPYY motifs. During their evolution, the *Ginger2*, *Copia *and *BEL *lineages might have lost the last two motifs.

Of the various *Gypsy *lineages shown in Figure 2, no lineage clusters with *Ginger1 *groups with significant bootstrap support. This does not preclude the possibility that *Ginger1 *groups arose from particular *Gypsy *lineages. It merely reflects the polytomy of *Gypsy *LTR retrotransposons [1]. The true ancestral lineages leading to *Ginger1 *may simply not be present in the available dataset, or they might have been lost in the evolutionary history, for example, due to the recombination process that produces the solo LTRs [24]. The chain of events that could lead to the hypothetical transformation of an LTR retrotransposon to a DNA transposon is also unclear. One simple possibility is that the TIRs of *Ginger1 *transposons were derived from two LTRs incidentally flanking the integrase region in opposite orientations: one would be the original 3' LTR of an LTR retrotransposon and the other coming from another LTR retrotransposon inserted upstream. The enzymatic mechanism underlying the excision of *Ginger1 *elements is also unknown (the same applies to *Polinton/Maverick *transposons). In the life cycle of LTR retrotransposons, integrases normally are responsible only for the integration process. However, *in vivo *data have shown that retrovirus integrases can reverse the initial strand-transfer reaction at the end of retroviral DNA [25]. Therefore, at least theoretically, *Ginger1 *DNA can be excised, but the excision process may not be as efficient as the excision of typical 'cut and paste' DNA transposons, since *Ginger1 *are small families and the copy numbers of individual elements per host are relatively low.

Despite the phylogenetic proximity of *Ginger2 *and *Polinton*/*Maverick *transposons in the phylogenetic tree (Figure 2), *Ginger2 *is less likely to be derived from *Polinton/Maverick *integrases, because *Polinton/Maverick *integrases lack the signature N-terminal C2H2 zinc finger. It cannot be resolved whether *Polinton/Maverick *integrases arose from *Ginger2 *or an LTR retrotransposon, because it is unclear whether the two fungal *Gypsy*-like lineages, clustering with *Polinton/Maverick *groups and retrovirus groups, respectively (Figure 2), represent some old *Gypsy *lineages or are just misplaced in the tree.

The Ulp1 protease C-terminal domain and OTU domain of *Ginger1 *transposases are also found in other transposable elements (Figure 1e, f). For example, Ulp1 domain is encoded in *Mutator *DNA transposons found in hydra [26], lancelet [27], *Arabidopsis thaliana *[28,29], maize [30], rice and *Cucumis melo *[31]; OTU domains are found in the proteins encoded by some *Helitron *DNA transposons in animals (Figure 1e) [32]. It has been suggested that Ulp1 proteases are functionally involved in the transposition process [29]. Interestingly, both the OTU and Ulp1 domains belong to the same C-protease family, and have similar functions in hydrolysis of ubiquitin or the small ubiquitin-like modifier (SUMO) protein [33,34]. Moreover, ubiquitinyl hydrolases are also found associated with other DNA transposases, such as protein XP_001314237.1 in protist *T. vaginalis *[35], which consist of transposase_11 domain (pfam01609) and Peptidase C19 (cd02657) domain. The latter domain participates in removing ubiquitin molecules from polyubiquinated peptides. These data imply that the ubiquitin pathway may be extensively involved in transposition. One attractive possibility is that both Ulp1 and OTU domains play an active role in the transposition processes. For example, they may activate the downstream factors in the DNA repair system after or during transposition, thereby minimizing the damage in the host genome. Indeed, all the major DNA repair pathways, damage avoidance mechanisms and checking responses are regulated somehow by ubiquitinylation, SUMOylation, or both [36].

## Conclusions

*Ginger *is a new superfamily of cut and paste DNA transposons coding for the transposase homologous to the integrase encoded by *Gypsy *LTR retrotransposons. The *Ginger *superfamily contains two distinct groups, *Ginger1 *and *Ginger2/Tdd*. Of the two groups, the *Ginger1 *transposases are more similar to *Gypsy *integrases. We also describe a number of host genes domesticated from *Ginger1 *TPase genes. This work takes a step towards finding the direct ancestors of an ancient DNA transposon recruited by a non-LTR retrotransposon to form the first LTR retrotransposon, and also raises the possibility of a new evolutionary pathway transforming a LTR retrotransposon to a cut and paste DNA transposon.

## Methods

### Data sources

Genomic sequences and mRNA sequences of various species were mainly taken from NCBI GenBank. *X. tropicalis *genome sequences (release 4.1 assembled scaffolds) were downloaded from Department of Energy (DOE) Joint Genome Institute (JGI) http://www.jgi.doe.gov/. For the phylogenetic analyses, integrase sequences were widely selected from the *Gypsy *Database (GyDB) http://gydb.uv.es/index.php/Main_Page[20], Repbase Update database http://www.girinst.org/repbase/index.html[37] and GenBank. Other transposable elements without specification of source in this paper are from the Repbase. Additionally, the sequences of TEs reported in this work have been deposited in Repbase with the same family names listed in Table 1 and Table 2.

### Sequence analysis

The consensus sequence of each *Ginger1 *or *Ginger2 *family, if possible, was rebuilt for analyses. The protein coding region of individual DNA transposons or host genes was either deduced from corresponding mRNA sequences, or manually predicted based on the sequence similarities between homologous proteins; exons and introns were determined accordingly. Multiple protein sequence alignments were carried out using MUSCLE [38] and were adjusted manually. Sequence alignments were edited and illustrated with BioEdit [39]. Logo representation of the TSD sequences was created by the WebLogo [40]. The phylogenetic tree was constructed using neighbor-joining (NJ) method and minimum evolution (ME) method (Poisson correction model, pairwise deletion, 1,000 bootstrap replicates) implemented in the MEGA4 software [41]. The gamma parameter for the phylogenetic tree was estimated using PhyMl [42].

## Competing interests

The authors declare that they have no competing interests.

## Authors' contributions

WB and JJ designed the initial research. WB prepared and processed the data. VVK contributed information on *Ginger2 *elements and suggested involvement of the ubiquitinylation and SUMOylation pathways in transposition. WB and JJ performed research and drafted the manuscript. All authors edited and approved the manuscript.

## Supplementary Material

Additional file 1 **Integrase alignments**. Integrase sequences alignment in the zinc finger domain and the DDE domain. Click here for file

Additional file 2 **Neighbor-joining phylogenetic tree**. Neighbor-joining phylogenetic tree is constructed using Poisson correction model, pairwise deletion, gamma parameter = 2, 1,000 bootstrap replicates. Click here for file

Additional file 3 **Protein sequence alignments**. Sequence alignments between host proteins, *Ginger1 *Tpases and *Gypsy *integrases. Click here for file
