# Supplementary material for: Ginger DNA transposons in eukaryotes and their evolutionary relationships with long terminal repeat retrotransposons
Source: Mob DNA. 2010 Jan 25;1:3. doi: 10.1186/1759-8753-1-3 (PMC2836005; doi:10.1186/1759-8753-1-3)
Supplement: Additional file 2 — Neighbor-joining phylogenetic tree. Neighbor-joining phylogenetic tree is constructed using Poisson correction model, pairwise deletion, gamma parameter = 2, 1,000 bootstrap replicates. [file 1759-8753-1-3-S2.PDF]

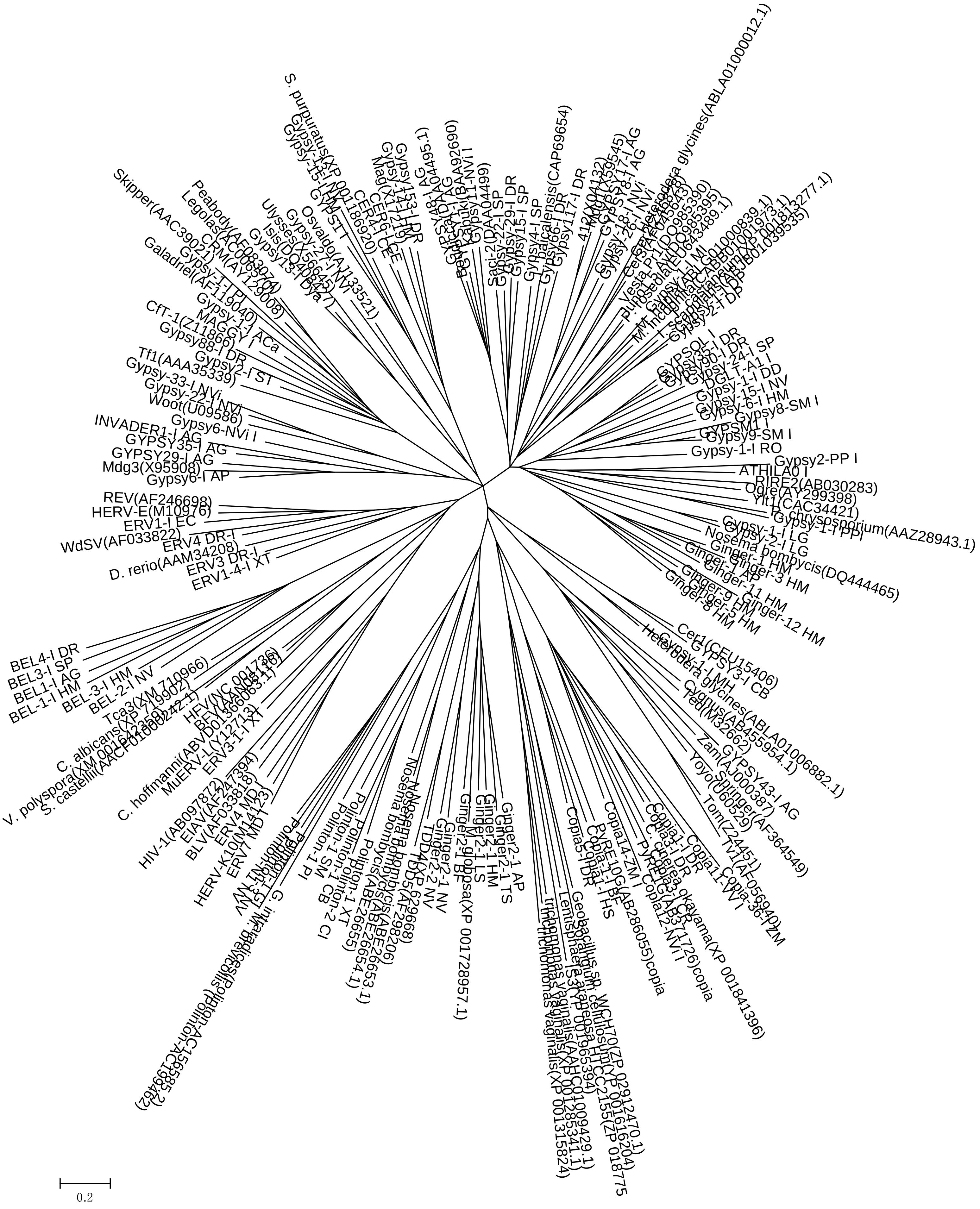

Gypsy-1-I RO
Gypsy2-PP I
ATHILA0 I
RIRE2(AB030283)
Ogre(AY299398)
Ylt1(CAC34421)
Nosema bombycis(DQ444465)
Cer1(CEU15406)
GYPSY3-I CB
Ted(M32662)
GYPSY43-I AG
Zam(AJ000387)
Yoyo(U60529)
Springer(AF364549)
Tom(Z24451)
Tv1(AF056940)
REV(AF246698)
HERV-E(M10976)
ERV1-I EC
WdSV(AF033822)
ERV4 DR-I
D. rerio(AAM34208)
ERV3 DR-I
ERV1-4-I XT
INVADER1-I AG
GYPSY35-I AG
GYPSY29-I AG
Mdg3(X95908)
Gypsy6-I AP
Gypsy6-NVi I
Woot(U09586)
Tf1(AAA35339)
Gypsy-33-I NVi
Gypsy-22-I NVi
Gypsy88-I DR
Gypsy2-I ST
CfT-1(Z11866)
MAGGY I
Galadriel(AF119040)
Skipper(AAC39021)
Peabody(AF083074)
Legolas(AC006370)
Osvaldo(AJ133521)
Ulysses(X56645)
Isis(DQ408427)
Saci-2(DAA04499)
Gypsy15-I SP
Gypsy4-I SP
Gypsy117-I DR
Gypsy-1-I MI
Gypsy-2-I DP
GYPSOL I
Gypsy35-I DR
Gypsy90-I DR
Gypsy-24-I SP
DGLT-A1 I
Gypsy-1-I DD
Gypsy-15-I NV
Gypsy-6-I HM
Gypsy8-SM I
GYPSM1 I
Gypsy9-SM I
Gypsy-1-I PPi
Gypsy-1-I LG
Gypsy-2-I LG
Ginger-3 HM
Ginger-1 HM
Ginger-1 AP
Ginger-11 HM
Ginger-12 HM
Ginger-9 HM
Ginger-5 HM
Ginger-8 HM
BEL4-I DR
BEL3-I SP
BEL1-I AG
BEL-1-I HM
BEL-3-I HM
BEL-2-I NV
Tca3(XM 710966)
HFV(NC 001736)
C. albicans(XP 719902)
V. polyspora(XM 001642239)
S. castellii(AACF01000242.1)
C. hoffmanni(ABVD01366063.1)
MuERV-L(Y12713)
ERV3-1-I XT
HIV-1(AB097872)
EIAV(AF247394)
BLV(AF033818)
ERV4 MD
HERV-K10(M14123)
ERV7 MDT
Polinton-1 PI
Polinton-1 SM
Polinton-1 CB
Polinton-1 XT
Polinton-2 CI
Ginger2-1 AP
Ginger2-1 TS
Ginger2-1 HM
Ginger2-1 LS
Ginger2-1 BF
Ginger2-1 NV
Ginger2-2 NV
TDD4
Copia5-I DR
Copia1-I HS
Copia14-ZM I
Copia3-I DR
Copia-36-I ZM
Copia-1-I NVi
0.2

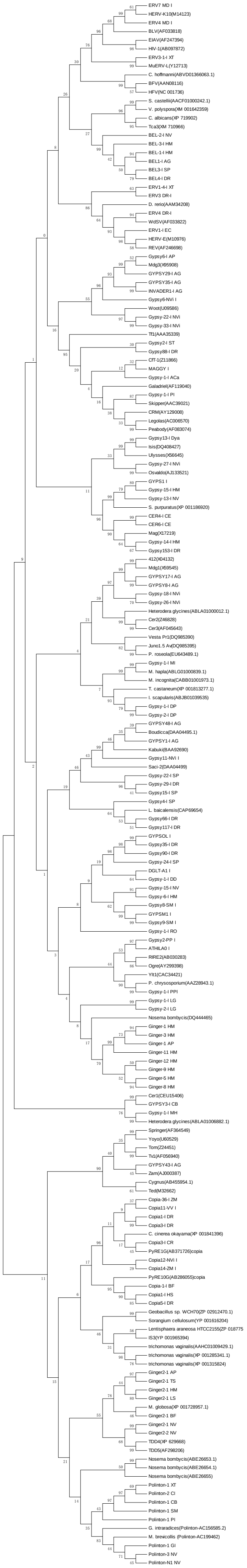

ERV7 MD I
HERV-K10(M14123)
ERV4 MD I
BLV(AF033818)
EIAV(AF247394)
HIV-1(AB097872)
ERV3-1-I XT
MuERV-L(Y12713)
C. hoffmanni(ABVD01366063.1)
BFV(AAN08116)
HFV(NC 001736)
S. castellii(AACF01000242.1)
V. polyspora(XM 001642359)
C. albicans(XP 719902)
Tca3(XM 710966)
BEL-2-I NV
BEL-3-I HM
BEL-1-I HM
BEL1-I AG
BEL3-I SP
BEL4-I DR
ERV1-4-I XT
ERV3 DR-I
D. rerio(AAM34208)
ERV4 DR-I
WdSV(AF033822)
ERV1-I EC
HERV-E(M10976)
REV(AF246698)
Gypsy6-I AP
Mdg3(X95908)
GYPSY29-I AG
GYPSY35-I AG
INVADER1-I AG
Gypsy6-NVi I
Woot(U09586)
Gypsy-22-I NVi
Gypsy-33-I NVi
Tf1(AAA35339)
Gypsy2-I ST
Gypsy88-I DR
Cft-1(Z11866)
MAGGY I
Gypsy-1-I ACa
Galadriel(AF119040)
Gypsy-1-I PI
Skipper(AAC39021)
CRM(AY129008)
Legolas(AC006570)
Peabody(AF083074)
Gypsy13-I Dya
Isis(DQ408427)
Ulysses(X56645)
Gypsy-27-I NVi
Osvaldo(AJ133521)
GYPS1 I
Gypsy-15-I HM
Gypsy-13-I NV
S. purpuratus(XP 001186920)
CER4-I CE
CER6-I CE
Mag(X17219)
Gypsy-14-I HM
Gypsy153-I DR
412(X04132)
Mdg1(X59545)
GYPSY17-I AG
GYPSY8-I AG
Gypsy-18-I NVi
Gypsy-26-I NVi
Heterodera glycines(ABLA01000012.1)
Cer2(Z46828)
Cer3(AF045643)
Vesta Pr1(DQ985390)
Juno1.5 Av(DQ985395)
P. roseola(EU643489.1)
Gypsy-1-I MI
M. hapla(ABLG01000839.1)
M. incognita(CABB01001973.1)
T. castaneum(XP 001813277.1)
I. scapularis(ABJB01039535)
Gypsy-1-I DP
Gypsy-2-I DP
GYPSY48-I AG
Boudicca(DAA04495.1)
GYPSY1-I AG
Kabuki(BAA92690)
Gypsy11-NVi I
Saci-2(DAA04499)
Gypsy-22-I SP
Gypsy-29-I DR
Gypsy15-I SP
Gypsy4-I SP
L. baicalensis(CAP69654)
Gypsy66-I DR
Gypsy117-I DR
GYPSOL I
Gypsy35-I DR
Gypsy90-I DR
Gypsy-24-I SP
DGLT-A1 I
Gypsy-1-I DD
Gypsy-15-I NV
Gypsy-6-I HM
Gypsy8-SM I
GYPSM1 I
Gypsy9-SM I
Gypsy-1-I RO
Gypsy2-PP I
ATHILA0 I
RIRE2(AB030283)
Ogre(AY299398)
Ylt1(CAC34421)
P. chrysosporium(AAZ28943.1)
Gypsy-1-I PPI
Gypsy-1-I LG
Gypsy-2-I LG
Nosema bombycis(DQ444465)
Ginger-1 HM
Ginger-3 HM
Ginger-1 AP
Ginger-11 HM
Ginger-12 HM
Ginger-9 HM
Ginger-5 HM
Ginger-8 HM
Cer1(CEU15406)
GYPSY3-I CB
Gypsy-1-I MH
Heterodera glycines(ABLA01006882.1)
Springer(AF364549)
Yoyo(U60529)
Tom(Z24451)
Tv1(AF056940)
GYPSY43-I AG
Zam(AJ000387)
Cygnus(AB455954.1)
Ted(M32662)
Copia-36-I ZM
Copia11-VV I
Copia1-I DR
Copia3-I DR
C. cinerea okayama(XP 001841396)
Copia3-I CR
PyRE1G(AB371726)copia
Copia12-NVi I
Copia14-ZM I
PyRE10G(AB286055)copia
Copia-1-I BF
Copia1-I HS
Copia5-I DR
Geobacillus sp. WCH70(ZP 02912470.1)
Sorangium cellulosum(YP 001616204)
Lentisphaera araneosa HTCC2155(ZP 018775
IS3(YP 001965394)
trichomonas vaginalis(AAHC01009429.1)
trichomonas vaginalis(XP 001285341.1)
trichomonas vaginalis(XP 001315824)
Ginger2-1 AP
Ginger2-1 TS
Ginger2-1 HM
Ginger2-1 LS
M. globosa(XP 001728957.1)
Ginger2-1 BF
Ginger2-1 NV
Ginger2-2 NV
TDD4(XP 629668)
TDD5(AF298206)
Nosema bombycis(ABE26653.1)
Nosema bombycis(ABE26654.1)
Nosema bombycis(ABE26655)
Polinton-1 XT
Polinton-2 CI
Polinton-1 CB
Polinton-1 SM
Polinton-1 PI
G. intraradices(Polinton-AC156585.2)
M. brevicollis (Polinton-AC199462)
Polinton-1 GI
Polinton-3 NV
Polinton-N1 NV
